# A Novel Architecture for Carbon Nanotube Membranes towards Fast and Efficient Oil/water Separation

**DOI:** 10.1038/s41598-018-25788-9

**Published:** 2018-05-09

**Authors:** Jayaprakash Saththasivam, Wubulikasimu Yiming, Kui Wang, Jian Jin, Zhaoyang Liu

**Affiliations:** 1 Qatar Environment and Energy Research Institute (QEERI), Hamad Bin Khalifa University (HBKU), Qatar Foundation, PO Box, 5825 Doha, Qatar; 2 Chemical Engineering Program, Texas A&M University at Qatar, Education City, Doha, 23874 Qatar; 3 School of Traffic & Transportation Engineering, Central South University, Changsha, 410075 China; 4 Suzhou Institute of Nano-Tech and Nano-Bionics, Chinese Academy of Sciences, Suzhou, 215123 China

## Abstract

Carbon nanotubes (CNT) are robust and proven as promising building blocks for oil/water separating membranes. However, according to classic fluid dynamic theory, achieving high permeation flux without sacrificing other membrane properties is a formidable challenge for CNT membranes, because of the trade-off nature among key membrane parameters. Herein, to relieve the trade-off between permeation fluxes, oil rejection rate, and membrane thickness, we present a new concept to engineer CNT membranes with a three-dimensional (3D) architecture. Apart from achieving high oil separation efficiency (>99.9%), these new oil/water separating membranes can achieve water flux as high as 5,500 L/m^2^.h.bar, which is one order of magnitude higher than pristine CNT membranes. Most importantly, these outstanding properties can be achieved without drastically slashing membrane thickness down to nanoscale. The present study sheds a new light for the adoption of CNT-based membranes in oil/water separation industry.

## Introduction

A large quantity of wastewater containing oil-in-water emulsions is generated on daily basis from various industries, including oil/gas exploration and production, oil product refinery and pharmaceutics, etc^1–3^. The separation of emulsified oil from wastewater is always a job that is tough and challenging, because of their tiny sizes of oil droplets^4^. Without proper treatment, the emulsified oils in these wastewaters will accumulate and pose a severe threat to the environment and human health^5,6^. Conventional techniques like gravity settling, centrifuges, and air flotation, all of which function on the basis of gravity difference, are not effective for the separation of oil-in-water emulsions^7,8^.

Filtration membranes, which function on the basis of “size-sieving” effect, are able to filter out a wide spectrum of pollutants with variable membrane pore sizes^9–11^. Membrane technology is gaining wider acceptance because it can produce effluents with acceptable qualities for environment discharge^12,13^. An ideal filtration membrane is one that has high permeation flux and high pollutant rejection rate, as well as low fouling propensity. A classical fluid dynamic theory for membrane design is the Hagen-Poiseuille equation:

1 $${\rm{J}}={{\rm{\varepsilon }}{\rm{\pi }}{\rm{r}}}_{{\rm{p}}}^{2}{\rm{\Delta }}p/8{\rm{\mu }}L$$

wherein, permeation flux J is directly proportional to the square of membrane pore radius r_p_ and inversely proportional to membrane thickness^14,15^. This fluid dynamic theory suggests that a formidable challenge lies in overcoming the trade-off relation between permeation flux and membrane thickness^16^. It is virtually impossible to improve permeation flux without sacrificing membrane thickness^17^.

Recently, a significant progress on oil/water separation has been made with the fabrication of ultrathin carbon nanotubes membranes^18–20^. These CNT membranes demonstrate effective separation of emulsified oil or water from oil/water mixtures with the outstanding advantages of ultrahigh flux and high separation efficiency. However, the ultrahigh permeation fluxes of these CNT membranes were achieved with severely sacrificing in term of membranes thickness (less than 70 nanometers), which inevitably increases handling complexity during membrane fabrication and sacrifices their mechanical strength for practical applications. Considering the efforts undertaken so far, it is still on the way to seek an ideal membrane towards fast and effective separation of emulsified oil/water mixtures with a negligible loss in other membrane properties.

In this study, a new concept is reported to engineer CNT membranes with three-dimensional (3D) architecture, to achieve fast and efficient separation of emulsified oil/water mixtures. These new membranes are constructed with CNT@MnO_2_ assembles (MnO_2_ nanorods conformally wrapped on CNTs). These tiny MnO_2_ nanorods are crucial in constructing the hierarchical 3D architecture, which is favorable for water readily passing through, at the same time maintaining sub-micron pore size to effectively sieve out emulsified oil droplets. Most importantly, these excellent properties can be achieved without the necessity of drastically reducing membrane thickness down to nanoscale. The new concept effectively relieves the formidable trade-off between permeation fluxes, oil rejection rate and the membrane thickness of CNT membranes. In addition, the new membrane exhibits good reusability with less oil fouling and easy cleanup. The present approach offers a practical route for a scalable fabrication of CNT-based membranes for fast and efficient oil/water separation.

## Results

**Figure 1:**
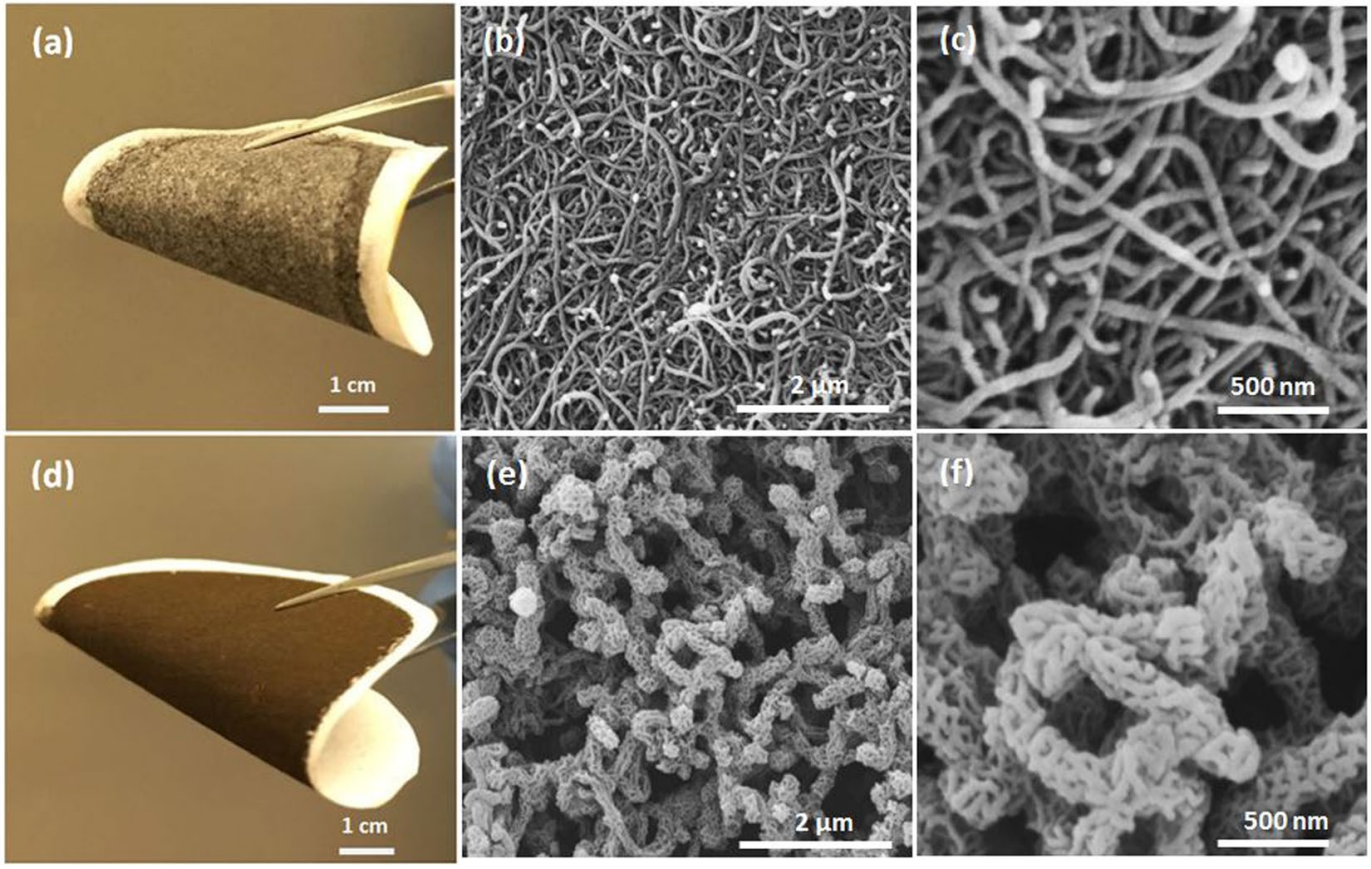
(**a**) Photo of membrane based on MWCNT/cellulose microfibers. (**b**) and (**c**) SEM images of MWCNT coated membrane at different magnifications. (**d**) Photo of membrane based on MWCNT-MnO_2_/cellulose microfibers. (**e**) and (**f**) SEM images of MWCNT-MNO_2_ coated membrane at different magnifications.

The depositions of MWCNT and MWCNT-MnO_2_ on cellulose microfiber substrates using vacuum filtration technique are shown in Fig. 1. It can be observed in subfigures 1(a) and 1(d) that both coated membranes are flexible and can be rolled and folded without cracks. Deposition of MWCNT on cellulose substrates leads to a very densely packed structure with a tight pore size as can be seen in Fig. 1(b,c). Uniform dispersion of MWCNT with an intertwined non-woven network can be also clearly observed. On the other hand, the 3D MWCNT-MnO_2_ membrane is more loosely packed and porous with a relatively larger pore size. It is can be observed that each of the MWCNT bundle surfaces was conformally wrapped and coiled with numerous short MnO_2_ nanorods, hence creating a 3D like porous structure. The MnO_2_ nanorods are approximately 50 μm in length and 15 nm in diameter. Also, it can be noted that the growth of MnO_2_ nanorods on MWNCTs is well distributed with no apparent agglomeration. The presence of MnO_2_ nanorods was confirmed using XRD analyses (Figure S3).

**Figure 2:**
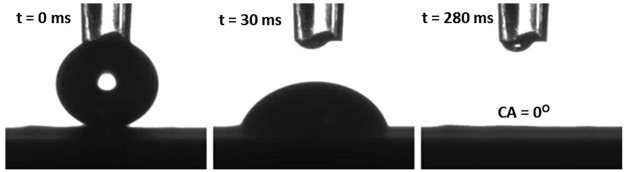
Quick spreading process of a water droplet of 4 µL into the MWCNT-MnO_2_ membrane.

Determination of surface hydrophilicity-hydrophobicity using contact angle measurements can provide a great insight into the antifouling properties of a membrane. In this study, water contact angle and underwater oil contact angle measurements were obtained to evaluate the antifouling properties of the MWCNT-MnO_2_ coated membrane. An average of three measurements of contact angle taken at random locations is reported here. The water contact angle readings for the pristine MWCNT and 3D MWCNT-MnO_2_ are 78 ± 0.5° and 0 ° respectively. This confirms that the addition of MnO_2_ nanorods has significantly improved the hydrophilicity of the membrane. The quick spreading (<1 s) of the water droplet on the surface of MWCNT-MnO_2_ coated membrane as shown in Fig. 2 further reaffirmed its super-hydrophilicity. Underwater oil contact angle measurement obtained using sunflower oil droplets for the 3D membrane is approximately as 152.3 ± 0.5^o^, thus renders it as an underwater superoleophobic membrane with a lower oil-fouling tendency when submerged in water. Additional measurements of underwater oil contact angle using different types of oils are included in the supporting information (Figure S4). The superhydrophilic- underwater superoleophobic property of the 3D membrane plays a significant role in lowering oil fouling tendency as the modified surface has great oil repelling tendency with low adherence and adsorption of oil droplets.

Performance of an oil-water separation membrane is generally assessed using two key parameters namely (i) oil rejection ratio and (ii) water flux. High oil rejection ratio can be easily achieved via the sieving effect using a membrane of finer pore size than emulsified oil droplets, which is typically in the range of 3 to 5 microns in diameter. For instance, conventional microfiltration and ultrafiltration membranes are capable of achieving high oil rejection rate due to their submicron pore size. However, these membranes suffer from low flux throughput and rapid oil fouling tendency. In this study, we have attempted to improve the water flux while maintaining high oil rejection ratio by developing a super hydrophilic 3D membrane using MWCNT and MnO_2_. These studies were conducted using emulsified sunflower oil (droplet size, d_50_ = 2.89 μm) where the oil-water emulsions were filtered using a dead end vacuum filtration set-up at a fixed pressure differential of 20 kPa.

**Figure 3:**
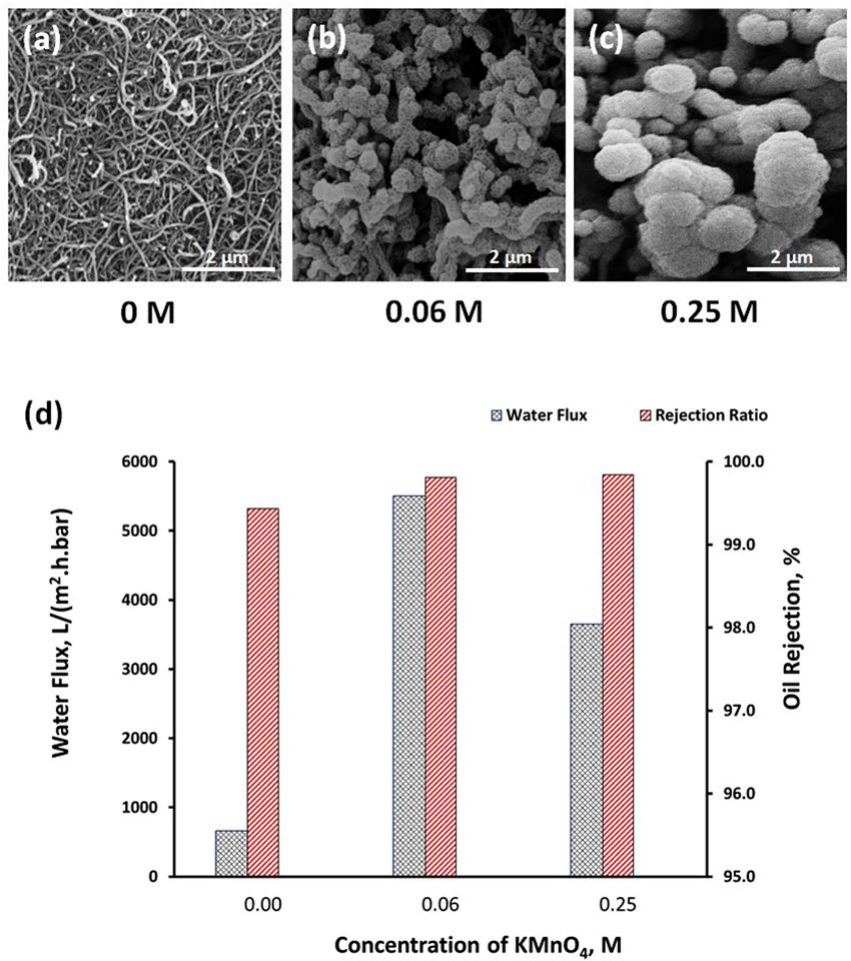
(**a**–**c**) SEM images of the membranes corresponding to KMnO_4_ concentrations of 0, 0.06 and 0.25 M respectively at a fixed CNT concentration. (**d**) Water flux and permeate oil content of sunflower oil-in-water emulsion feed using similar membranes.

**Figure 4:**
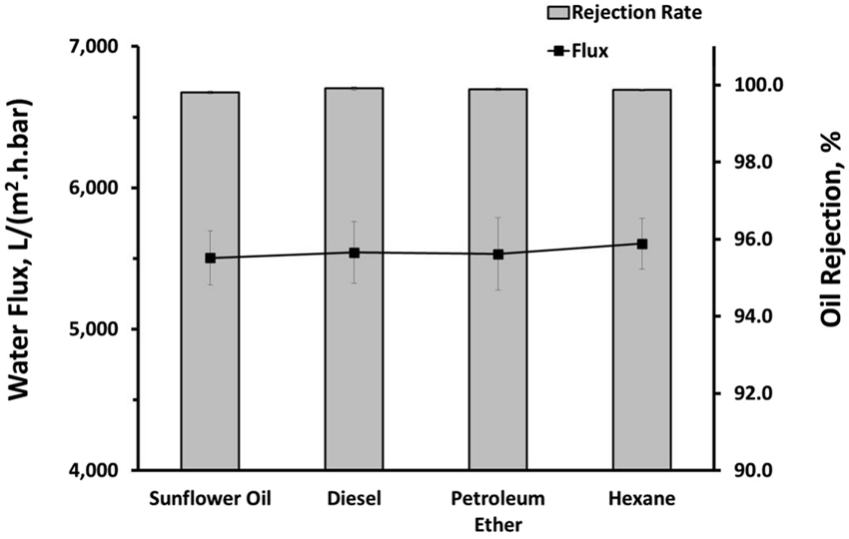
Permeate water flux and oil rejection ratio of different emulsified oil feeds filtrated using MWCNT-MnO_2_ membrane.

From Fig. 3, it can be seen that both oil rejection ratio and water flux of MWCNT membrane increased with the addition of KMnO_4_ as MnO_2_ precursor. The redox reaction between KMnO_4_ and MWCNT produces super-hydrophilic MnO_2_ nanorods that coil and wrap the surface of the MWCNT as shown in SEM observations. It can be observed that the oil rejection rates for all the cases were very high. The oil rejection ratio of the pristine MWCNT is approximately 99.4%. Addition of KMnO_4_ that facilitates the formation of superhydrophilic MnO_2_ via the redox reaction has improved the oil rejection ratio close to 100%. From the perspective of oil rejection ratio, this trivial improvement clearly did not warrant the usage of MWCNT-MnO_2_. However, the real potential of this 3D membrane can be realized by evaluating the water flux data. From Fig. 3, it is shown that water flux of the membrane improved with the addition of the KMnO_4_. In comparison with the pristine MWCNT membrane, the water flux improved from approximately from 660 L/(m^2^.h.bar) to over 5,500 L/(m^2^.h.bar) when the concentration of KMnO_4_ was increased from 0 to 0.06 M. The enhanced permeability of the membrane is due to superhydrophilicity of MnO_2_ that consists of many –OH functional groups^21^. Apart from the superhydrophilic surface property, the improvement of the flux can be also attributed to the 3-dimensional porous integrated network structure of the 3D membrane. The MWCNT-MnO_2_ membrane is less densely packed if compared with MWCNT membrane as shown in both Figs 1 and 3. It is evident from these results that water flux of CNT based membrane can be significantly increased without comprising the oil rejection ratio using MnO_2_ as nanofillers. Further addition of KMnO_4_ has a detrimental effect on the flux. This is primarily due to change in nanostructure of MnO_2_ from nanorods to nanospheres as shown in Fig. 3(b,c) when the KMnO_4_ concentration was increased from 0.06 M to 0.25 M. Excessive addition of KMnO_4_ led to formation of agglomerated MnO_2_ nanospheres on the surface of the MWCNT that reduce the porosity and pore size of the membrane, hence effectively reducing the permeability of the 3D membrane. For all the subsequent filtration performance studies, the 3D MWCNT-MnO_2_ membrane membranes were prepared using the optimized KMnO_2_ concentration of 0.06 M. The synthesized membranes were also tested against different feeds of emulsified oil, namely (i) diesel (ii) petroleum ether and (iii) hexane. The rejection ratios were close to 100% for all the different feeds as shown in Fig. 4. The permeate flux obtained for these feeds were also very close to the flux (e.g. approximately 5,500 ± 250 L/(m^2^.h.bar)) achieved for the emulsified sunflower oil. These results can be well correlated with the underwater oil contact angle readings shown in the supporting information (Figure S4). For all the four different feeds, the oil contact angle readings were over 150°, thus clearly indicating the underwater superoleophobicity of the membrane.

**Figure 5:**
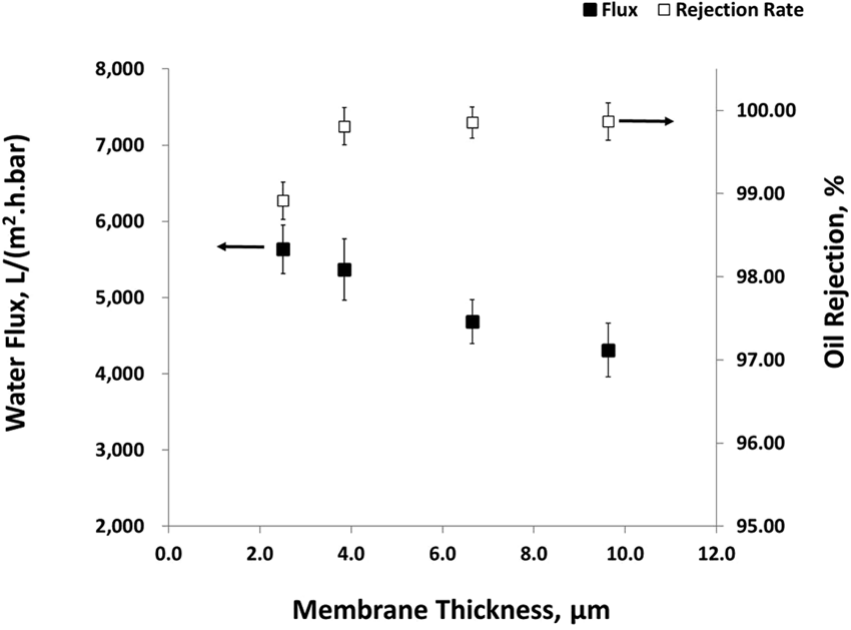
Permeate water flux and oil rejection ratio as a function of 3D membrane thickness.

Permeate water flux and oil rejection ratio as a function of membrane thickness is shown in Fig. 5. As the thickness was increased from 2.5 µm to 10 µm, water flux of the composite membrane was not dramatically reduced, and remain in the range of between 5,634.4 L/m^2^.h.bar and 4,311.7 L/m^2^.h.bar. This is starkly in contrast to previously published studies^18–20^, in which the membrane has to be ultrathin (less than 70 nanometers) to achieve such a high flux. Ultrathin membrane suffers from poor mechanical strength and requires complicated membrane handling procedure during practical application; therefore hampering the wide adoption of CNT membranes in industries. In our study, we circumvent this problem by designing a hierarchical 3D structure for CNT membranes, which minimizes the necessity to drastically reduce the membrane thickness, and thus avoids the handling of delicate ultrathin membranes during the membrane fabrication and application processes, and enhance the potential for CNT membranes in practical applications.

**Figure 6:**
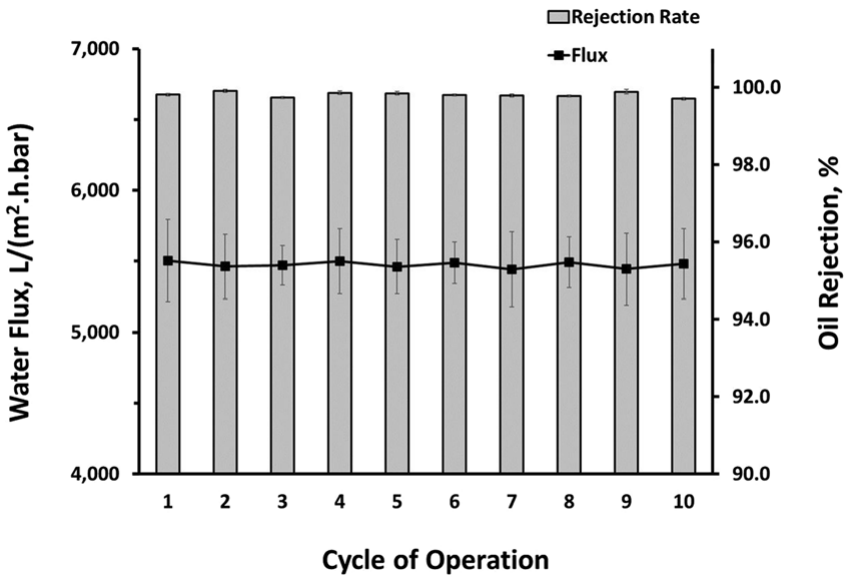
Permeate water flux and oil rejection ratio of the MWCNT-MNO_2_ over period of 10 operating cycles.

The anti-fouling performance of the 3D membrane was evaluated over ten cycles of operations. In each cycle, 150 ml of emulsified sunflower oil at a concentration of 1% v/v was filtered using the 3D membrane. The membrane was flushed with 50 ml of hot water (50–60 °C) in between the filtration cycles. As shown in Fig. 6, the 3D membrane was capable of maintaining almost 100% oil rejection ratio throughout the entire cycles of operations. As for the flux, the membrane was able to achieve a consistent filtration capacity (average flux = 5,475 ± 208 L*/(*m^2^.h.bar)). The fact that the permeability of the membrane was easily recovered after a simple hot flush demonstrates that the membrane is less prone to irreversible fouling. The underwater superoleophobic and superhydrophilic nature of the 3D membrane prevents oil droplets from firmly attaching to its surface. Due to these unique surface properties, the reversible fouling layer of the membrane can be easily removed using simple hydraulic and thermal cleaning.

## Discussion

Despite the numerous advantages of CNT, their applications in membrane filtration systems are somehow limited due to the trade-off between the key properties: permeate flux and membrane thickness. The oleophilic surface property of CNT is also prone to oil fouling and deposits. The state of the art to achieve high permeation flux for CNT membranes is to make membrane thickness at nanoscale, the practice of which inevitably hampers the wide application of CNT membranes, due to the complex fabrication and delicate handling for these ultrathin membranes. In this study, we have improved the flux and anti-fouling properties of CNT based membrane by conformably growing superhydrophilic MnO_2_ nanorods on the surface of CNT. The conformal coating of MnO_2_ is crucial in providing 3D inter-connected channels for water passage, as well as maintaining sub-micron pore size for oil rejection. The 3D membrane also displayed exceptional anti-fouling features due to the presence of MnO_2_ nanorods that extensively altered the surface property of CNT from oleophilic to underwater superoleophobic. This study clearly demonstrates the advantages of using the CNT-MnO_2_ membrane to filter emulsified oil. Most importantly, all the advantages were achieved without significantly sacrificing the membrane thickness. In addition, the new membranes can be easily cleaned up with an environmental-friendly practice, due to its superior anti-fouling property. These findings shed a new light on extending the practical application of CNT membranes in oil-water separation industries.

## Methods

### Preparation of MWCNT-MnO_2_ membrane

10 mg of MWCNT (Sigma Aldrich: 6–9 nm OD *×* 5 μm L, >95% carbon) was dispersed in 50 ml 1% SDS solution using probe sonicator for 10 minutes. The homogenized solution was then centrifuged at 4500 × g for 10 minutes to remove large nanotube bundles and impurities such as amorphous carbon, graphitic particles and etc. The supernatant of the solution was decanted and adjusted to a final concentration of 40 mg/L based on the dried weight of MWCNT filtered on a membrane. 1 ml of the 40 mg/L CNT was then reacted with 9 mL acidified KMnO_4_ for 15 minutes at 60 °C in a temperature regulated water bath^22^. The optimal concentration of KMnO_4_ that is required to develop a high oil rejection rate and high water flux MWCNT-MnO_2_ membrane is described in the Results section of this manuscript. The reacted solution was diluted with deionized water prior to deposition on a 2.5 µm cellulose filter (Whatman grade 42 ashless filter papers) using a vacuum filtration device. The coated filter was washed multiple times using deionized water to remove excess permanganate and SDS solutions and then used for the emulsified oil separation studies.

### Oil-in-water emulsion preparation

Oil-in-water emulsion feed was prepared by sonicating 10 mL of sunflower oil in 90 mL of deionized water using an ultrasonic bath (8510E-DTH, Branson) for five minutes. This solution was then diluted using 900 mL deionized water to a final stock concentration of 1% v/v. Additional details related to the oil-in-water emulsion can be obtained in the Figures S1 and S2 included in the supporting information.

### Membrane Characterization

Surface morphological properties of the synthesized membranes were examined using environmental scanning electron microscope (FEI Quanta 400). Surface wetting behaviors of the synthesized membranes were measured using an advanced goniometer (rame-hart Model 500). X-ray diffraction spectra of the MnO_2_ were examined using Rigaku Ultima IV multipurpose X-ray diffractometer.

### Filtration Performance Testing

A vacuum filtration device (Nalgene) with an effective membrane area of 11.3 cm^2^ was used to the evaluate oil rejection ratio and flux of the MWCNT-MnO_2_ membranes at a fixed differential pressure of 20 kPa. Oil rejection ratio was calculated as follows:-

2 $$RR=(1-\frac{{C}_{p}}{{C}_{f}})\times 100 \% $$

where $RR$ represents rejection rate (%), *C*_*p*_ and *C*_*f*_ *d*enotes the Total Organic Carbon (TOC) values in the permeate and feed solution respectively. TOC values were measured using Total Organic Carbon Analyzer, (TOC-L, Shimadzu). Permeate flux of the membrane was calculated by measuring the permeate volume at a fixed filtration time and differential pressure of 20 kPa.

## Electronic supplementary material

Supplementary Information
