# Supplementary material for: A Novel Architecture for Carbon Nanotube Membranes towards Fast and Efficient Oil/water Separation
Source: Sci Rep. 2018 May 9;8:7418. doi: 10.1038/s41598-018-25788-9 (PMC5943308; doi:10.1038/s41598-018-25788-9)
Supplement: Supplementary file 1 — Supplementary Information [file 41598_2018_25788_MOESM1_ESM.pdf]

Supplementary Information

A Novel Architecture for Carbon Nanotube Membranes towards Fast and Efficient Oil/water Separation

Jayaprakash Saththasivam[1], Wubulikasimu Yiming[2], Kui Wang[3], Jian Jin[4] & Zhaoyang Liu[1*]

[1]Qatar Environment and Energy Research Institute (QEERI), Hamad Bin Khalifa University (HBKU), Qatar Foundation, PO Box 5825, Doha, Qatar
[2]Chemical Engineering Program, Texas A&M University at Qatar, Education City, Doha 23874, Qatar
[3]School of Traffic & Transportation Engineering, Central South University, Changsha 410075, China
[4]Suzhou Institute of Nano-Tech and Nano-Bionics, Chinese Academy of Sciences, Suzhou 215123, China
** Corresponding Author: E-mail address: zhliu@hbku.edu.qa (Zhaoyang Liu)*

(1) Oil in Water Emulsion Photos

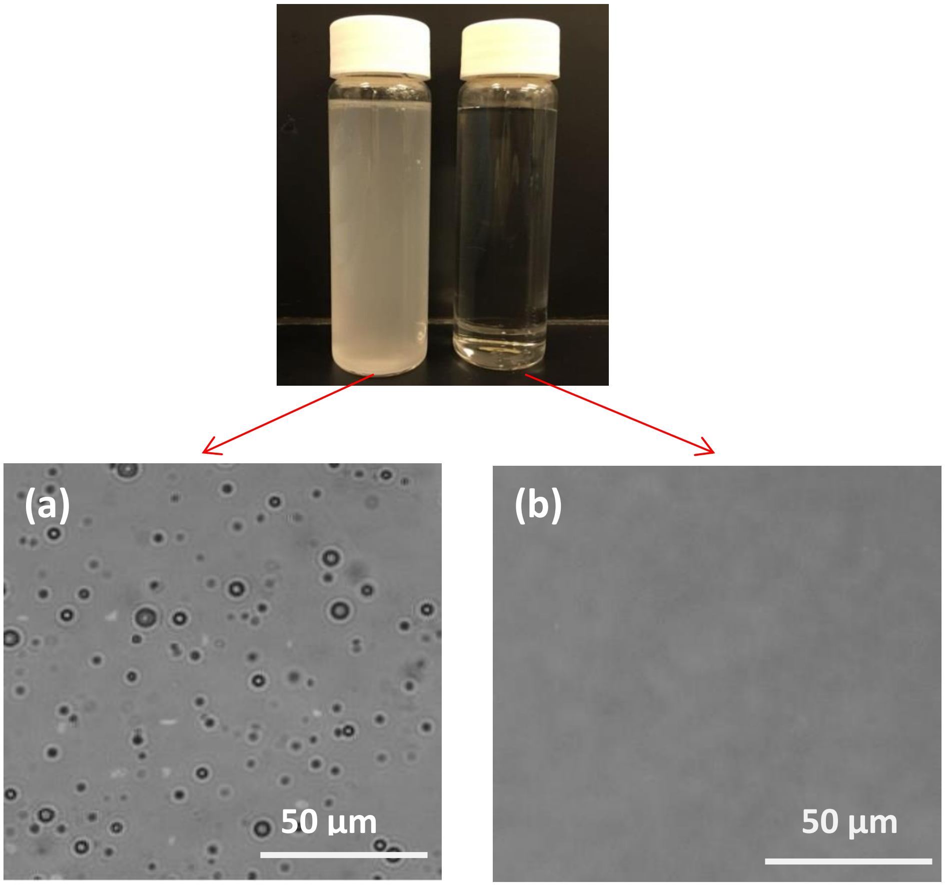

Figure S1: Photos and microscope images of (a) Sunflower oil-in-water emulsion feed and (b Filtrate of of the feed using MWCNT/MnO_2 membrane.

The oil-in-water emulsion feed and permeate of the hybrid membrane were observed using an optical microscopy Olympus IX73 (Shinjuku, Tokyo, Japan). The presence of micron size oil droplets can be clearly seen in the feed of the oil-in-water feed as shown in Figure S1. On the other hand, no visible droplet can be observed in the permeate of the MWCNT/MnO_2 membrane.

(2) Particle size distribtion of the Oil-in-Water emulsion

Figure S2 shows the size distribution of oil droplets in the emulsion feed that was measured using JORIN- ViPA B HiFlow (Leicestershire, UK) visual process analyzer. The statistical analysis of the droplet

distribution data revealed that the mean diameter of the droplets was around 3.62 μm with a d_{50} and d_{90} of 2.89 μm and 5.98 μm respectively. Preparation of subsequent batches of emulsion also displayed similar results, thus indicating that the proposed emulsion preparation method is capable of producing consistent droplet size distribution.

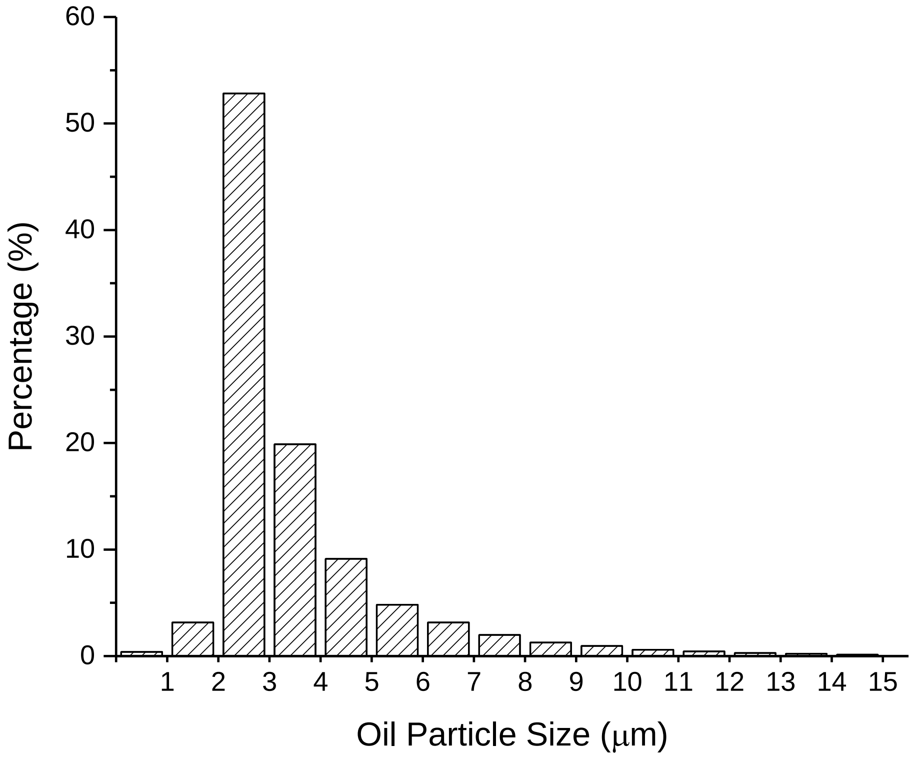

Figure S2: Sunflower oil droplet size distribution in as-prepared oil-in-water emulsions.

(3) XRD Analysis

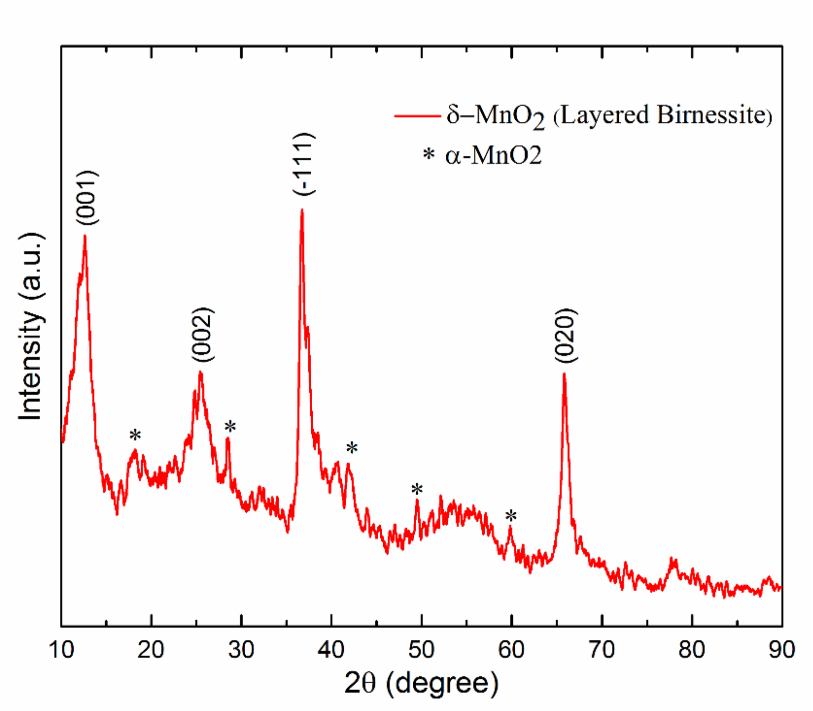

Figure S3: XRD profile of MWCNT/MnO_2 membrane.

A Rigaku Ultima IV multipurpose X-ray diffractometer equipped with Cu Ka radiation (λ =1.540 60 Å) and fixed monochromator was applied to collect the powder-XRD pattern. The acceleration voltage was 40 kV and the current was 40 mA. The XRD patterns were collected using step scan mode from 2theta (2θ) angle 10 to 90 degree with step size of 0.02 degree and scan speed of 1 second/step. The XRD data then analyzed using the Rigaku PDXL2 powder diffraction analysis software. Figure S3 shows powder XRD pattern of the as prepared MnO_2 nanorods. There are four major peaks at 12.7°, 25.4°, 36.7°, 65.8°, which correspond to the (001), (002), (-111) and (020) planes of the monoclinic Birnessite-type layered manganese oxide crystal (JCPDS No. 80-1098), also denoted as δ-MnO_2 in various studies[1,2]. In addition, it can be seen that there are number of low intensity peaks present, which are attributed to tetragonal α-MnO2 phase (JCPDS No. 44-0141).

(4) Underwater Oil Contact Angle measurement

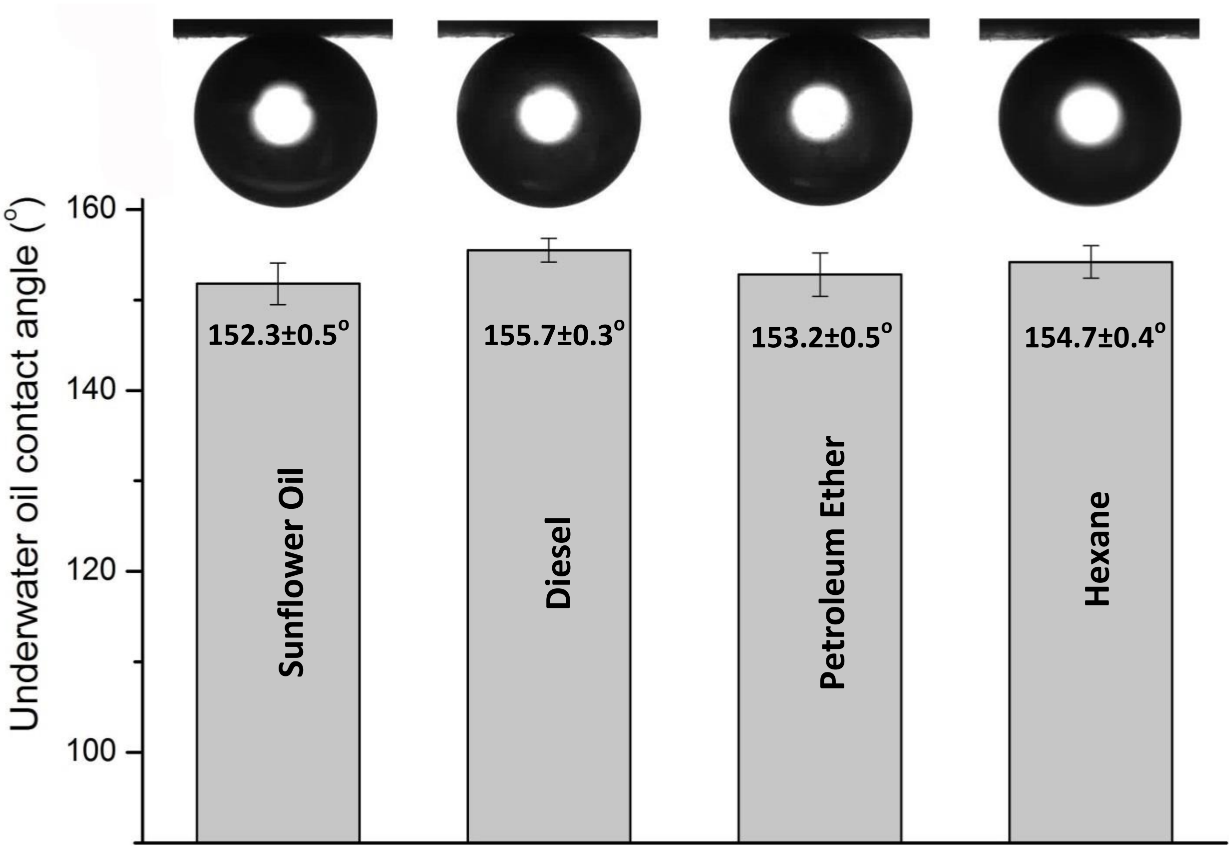

Figure S4: Underwater oil contact angles of different oils for MWCNT-MnO_2 membrane

As shown in Figure S4, the synthesized MWCNT-MnO_2 membrane displayed an impressive underwater oil contact angles of more than 150^0 for all the four different types of oils (e.g. sunflower oil, diesel, petroleum ether and hexane) used in this study. This indicates that the membrane is highly underwater superoleophobic where oil droplets will have minimal adherence to the membrane surface, hence significantly minimize the fouling potential of the membrane.

(5) Top surface pore size distribution of MWCNT/MnO_2 membrane

The pore size distribution of the hybrid MWCNT/MnO_2 membrane as shown in Figure S5 was estimated by analyzing SEM images using an image processing software (Image-J, National Institutes of Health, Bethesda, Maryland, USA). Statitical analyses of the processed images showed that mean pore of the membrane is approximately 0.35 micron.

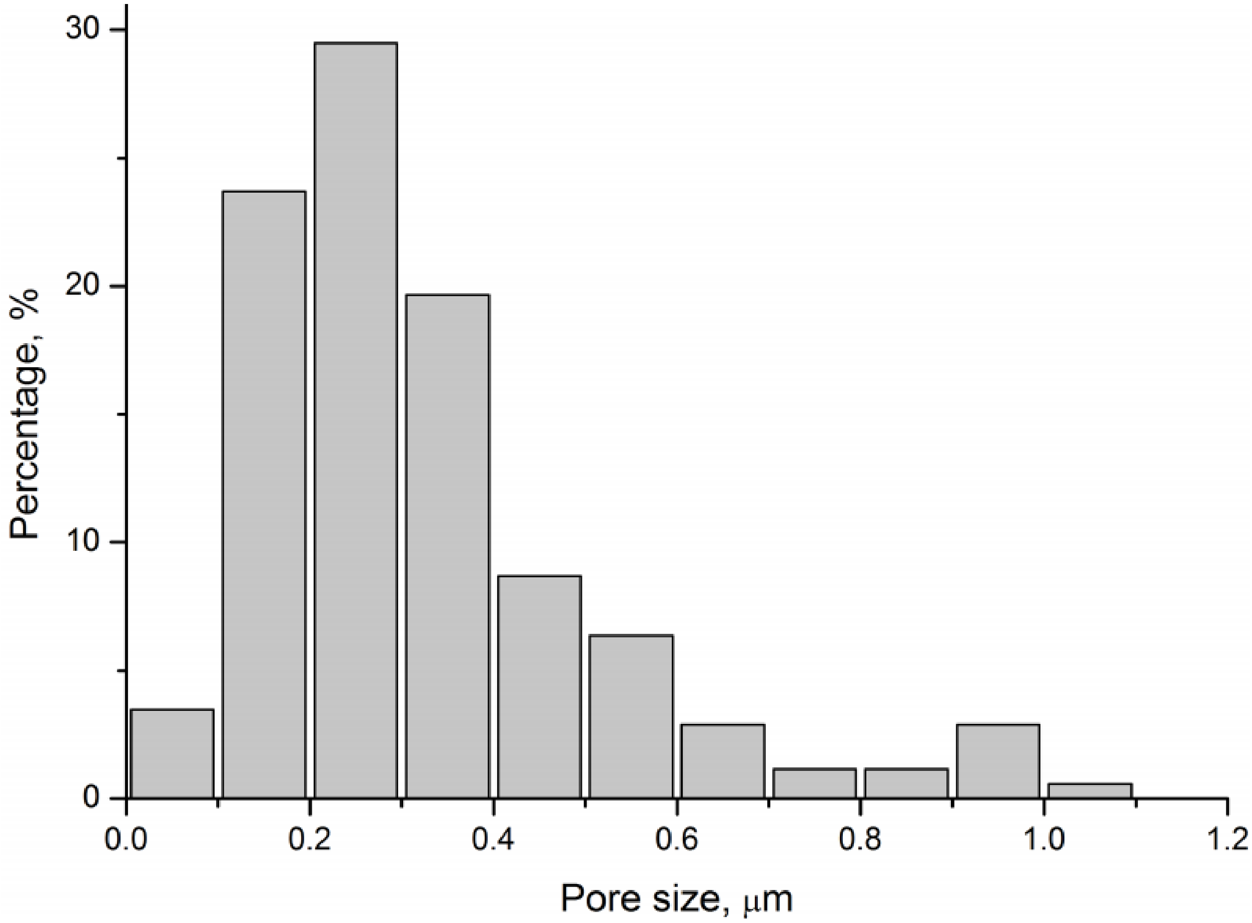

Figure S5: Top surface pore size distribution and mean pore size of the MWCNT/MnO_2 membrane

(6) Coating thickness of MWCNT/MnO_2 membrane

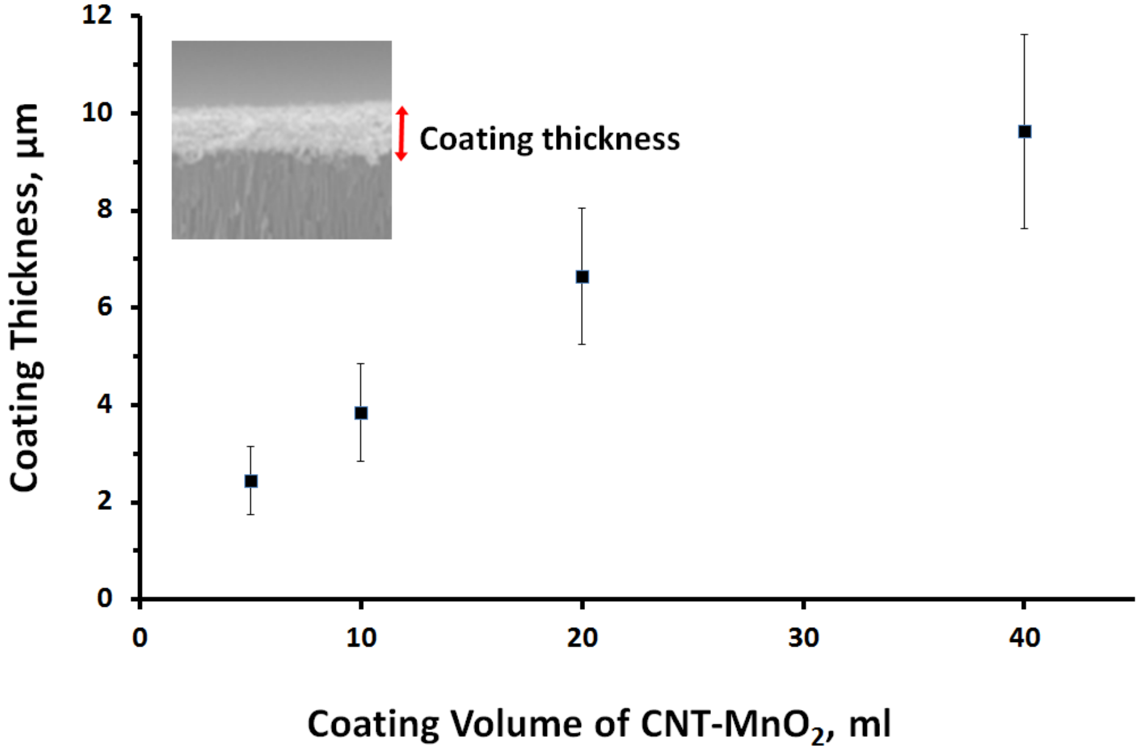

Figure S6: Coating thickness with respect to different deposition volume of MWCNT-MnO_2.

The coating thickness of MWCNT-MnO_2 with respect to the deposition volume is estimated using SEM cross-section images. Figure S6 shows the average thickness measured at three different locations of the membrane against the coating volume. It can be seen that the membrane thickness increases linearly as the deposition volume increases. However, it has to be emphasized that the standard deviation of the coating thickness is bigger at higher deposition volume. This could be due to agglomeration between the nanoparticles that led to non-uniform dispersion during the coating process.

(7) Porosity and Pores size measurement

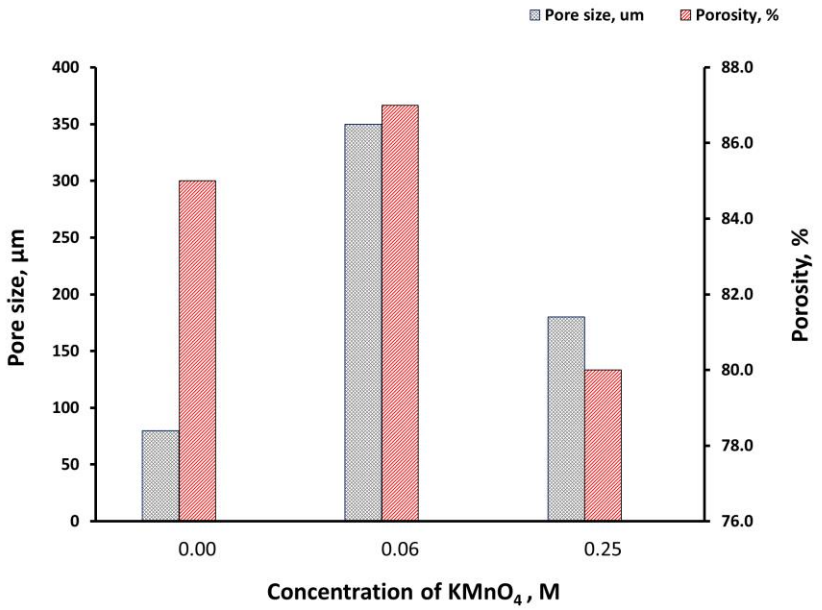

Fig. S7. Mean pore sizes and porosities of the 3D membranes at different concentrations of $KMnO_4$ precursor.

The mean pore size of the membranes were obtained by analyzing the SEM images using an image processing software (Image-J, National Institutes of Health, Bethesda, Maryland, USA). The porosity measurement was measured using gravimetric method[3].

(8) Mechanical properties

Burst test was used to investigate the mechanical response of the membranes. Figure S8 exhibits pressure - deflection response of the MWCNT-MnO_2 membrane comparing with that of the cellulose membrane. As shown in Figure S8, the burst pressure and breakage deflection of the MWCNT-MnO_2 membrane were higher than those of the cellulose membrane. The higher burst pressure and breakage deflection of the MWCNT-MnO_2 membrane are mainly due to the compact intertwined structure caused by the integration of MnO_2 nanorods modified MWCNTs with cellulose microfibers. The effective network between the MnO_2 nanorods modified MWCNTs and cellulose microfibers benefited stress transfer delaying the facture of the membrane, and consequently resulting in higher mechanical properties.

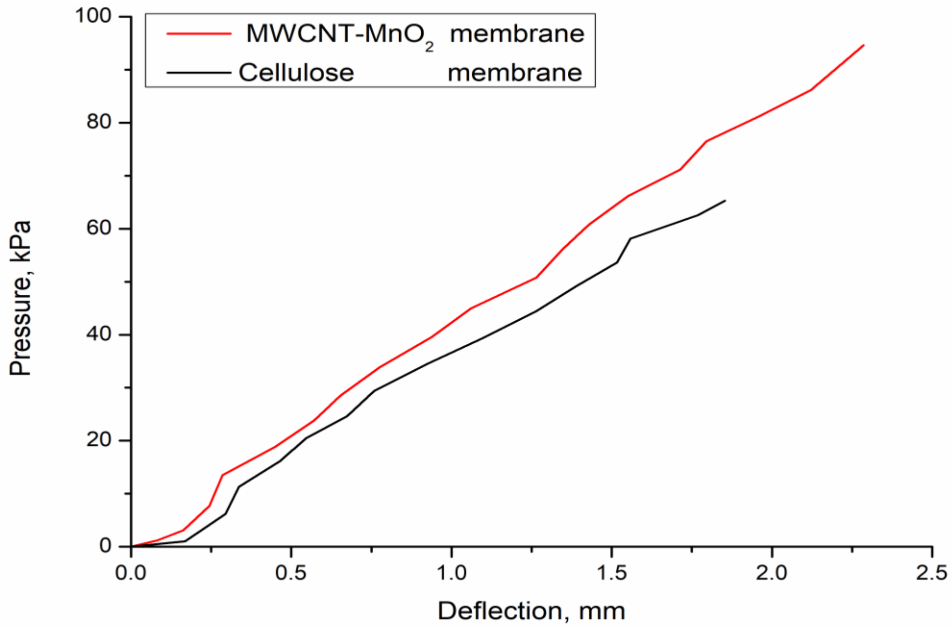

Figure S8: Pressure - deflection behaviors of the MWCNT-MnO2 membrane and cellulose membranes obtained by burst test.

References

1. Portehault, D., Cassaignon, S., Baudrin, E. & Jolivet, J.-P. Structural and morphological control of manganese oxide nanoparticles upon soft aqueous precipitation through MnO4−/Mn2+ reaction. *J. Mater. Chem.* **19,** 2407 (2009).

2. Zahoor, A. *et al.* A comparative study of nanostructured α and δ MnO2 for lithium oxygen battery application. *RSC Adv.* **4,** 8973 (2014).

3. Li, X. *et al.* A novel profiled core–shell nanofibrous membrane for wastewater treatment by direct contact membrane distillation. *J. Mater. Chem. A* **4,** 14453–14463 (2016).
